# Fungicide-Saving Potential and Economic Advantages of Fungus-Resistant Grapevine Cultivars

**DOI:** 10.3390/plants12173120

**Published:** 2023-08-30

**Authors:** Birgit Eisenmann, Chantal Wingerter, Marc Dressler, Christine Freund, Andreas Kortekamp, Jochen Bogs

**Affiliations:** 1 Horticulture and Rural Development, State Education and Research Center of Viticulture, 67435 Neustadt, Germany; birgit.eisenmann@dlr.rlp.de (B.E.); chantal.wingerter@dlr.rlp.de (C.W.); andreas.kortekamp@dlr.rlp.de (A.K.); 2 Department of Marketing and Entrepreneurship, Ludwigshafen University of Business and Society, 67059 Ludwigshafen, Germany; marc.dressler@hwg-lu.de; 3 Department of Life Sciences and Engineering, Bingen Technical University of Applied Sciences, 55411 Bingen, Germany

**Keywords:** disease resistance, downy mildew, powdery mildew, grapevine, fungus-resistant cultivars, *Vitis vinifera*, *Plasmopara viticola*, *Erysiphe necator*, plant protection

## Abstract

The high susceptibility of European grapevine cultivars to downy mildew (DM) and powdery mildew (PM) causes the intensive use of fungicides. Fungus-resistant cultivars (FRCs) with different resistance (*R*) loci have been bred and could play an important role in reducing plant protection treatments (PPTs). However, little information is available about the extent to which PPTs can be reduced in the field through the use of FRCs and the associated economic advantages. In this study, different strategies with reduced PPTs on FRCs were tested in field experiments. The results demonstrated that the number of PPTs can be reduced by 60 to 90%, resulting in reductions in applied copper and sulfur by 52 to 79% through the use of FRCs compared with susceptible cultivars, without affecting grape or plant health. The saving potential varied among years, depending on the type of *R* loci and climatic conditions. Furthermore, this study highlights that completely omitting PPTs in the cultivation of FRCs can result in PM or DM infections and possible loss of yield and fruit quality. In addition to the field experiments, a two-year observation of the performance of FRCs in commercial vineyards was undertaken, which highlighted not only the significant reduction in PPTs but also the financial savings that can be achieved through the use of FRCs.

## 1. Introduction

Grapevine is one of the most important fruit crops worldwide and is based almost exclusively on the cultivation of traditional *V. vinifera* cultivars for grape production [1]. These grapevine cultivars are highly susceptible to the pathogens that cause two of the most prevalent grapevine diseases worldwide, grapevine downy mildew (DM) and grapevine powdery mildew (PM). DM is caused by the obligate biotrophic endoparasite *Plasmopara viticola* (Berk. & M.A. Curtis) Berl. & de Toni, while PM is caused by the obligate biotrophic ectoparasite *Erysiphe necator* Schweinitz Braun & Takamatsu. Both pathogens are able to infect all green parts of grapevines, causing significant reductions in yield and berry quality [2,3,4]. The two mildew pathogens differ in their optimal infection conditions, primarily because of different optimal temperatures and the availability of water, which is crucial for the infection process of *P. viticola,* whereas precipitation limits the development of *E. necator*, for which a distinct level of humidity is mandatory [5]. Since the infection and development of the pathogens depend on different climatic conditions, the timing, frequency, and durableness of plant protection treatments (PPTs) is of high importance to prevent reductions in yield and grape berry quality.

Due to the high susceptibility of *V. vinifera* cultivars to DM and PM, the grape production is heavily dependent on the use of fungicides, irrespective of the cultivation method applied. Although only 5% of the European agricultural area is devoted to grapevine cultivation, approximately 70% of all fungicides applied in Europe are used to control fungal diseases in viticulture [6]. It is therefore evident in this agricultural sector, more than in any other, that the use of fungicides should be significantly reduced. Since the extensive use of plant protection products (PPPs) may be associated with adverse impacts on the environment and operators, the European Union has implemented the Green Deal, pursuing the goal of reducing their use by 50% by 2030 [7,8,9,10,11]. Even though copper-containing PPPs are currently the only permitted and reliably effective products to fight against DM in organic viticulture, they are classified as substitution candidates according to EU regulation 2015/408 and should be replaced, since copper accumulates in the upper soil layer and can negatively affect invertebrates and soil microorganisms [12,13,14]. However, complete omission of copper in organic production can result in total crop losses when environmental conditions favor pathogen development [15].

Various approaches to improve crop protection have been developed including technical support, such as antidrift and recycling technology or sensor-based systems. In addition to these technical improvements, a number of breeding programs have been applied for the introgression of resistance (*R*) loci from wild North American and Asian Vitis species into *V. vinifera* that confer resistance to mildew pathogens, resulting in new fungus-resistant cultivars (FRCs) [16,17]. These *R* loci confer resistance to *Plasmopara viticola* (*Rpv*) and resistance to *Erysiphe necator* (*Ren*, *Run*).

Although different quantitative trait loci (QTL) associated with resistance against DM and PM are now known, most FRCs grown in Europe rely on a single major *R* locus to resist DM (*Rpv3*) and PM (*Ren3*) [18]. The *Rpv3* locus was first identified in the interspecific hybrid cultivar ‘Regent’ and described in more detail in the hybrid cultivar ‘Bianca’ [19,20,21,22]. Further characterization of the previously identified *Rpv3*-locus revealed the existence of a number of allelic forms of this locus, i.e., *Rpv3-1*, *Rpv3-2* and *Rpv3-3,* which all mediate resistance to DM [22,23,24]. Other new cultivars with *Rpv10*-, *Rpv12-,* or *Run1*-mediated mildew resistance have also been introduced but are still cultivated to a much lower extent in Germany [25,26,27]. Although cultivars with different *R* loci against DM are now available, the FRCs currently in cultivation rely on one major R locus (*Ren3*) for resistance to PM. However, further cultivars have been released onto the market with the *Run1* locus as an additional PM *R* locus [25,28].

Monogenetically based resistance bears general the risk of pathogens developing that are able to overcome these single *R* loci [29,30]. Indeed, several studies have revealed that *P. viticola* isolates emerged in Europe that are able to overcome the resistance mediated by the *Rpv3* locus [31,32,33,34]. Moreover, in North America, at least one *E. necator* isolate is able to overcome *Run1*-mediated resistance [35]. As a perennial crop, the durability of resistance is much more important for grapevines, as annual crops are cultivated in sequence with other crops, limiting the development and propagation of host-specific pathogens [27]. Therefore, increasing attention is now being paid to the breeding of new cultivars containing multiple *R* loci against both DM and PM; in recent years, the first FRCs with polygenic resistance have been registered [26,28]. Despite the obvious advantages of using FRCs for wine production, these new cultivars currently represent only 2.6% of the vineyard area in Germany [25]. The current reluctance to adopt these cultivars can be attributed to a weak awareness among consumers, which results in high marketing costs, and a low level of experience in winemaking. Furthermore, information on plant protection strategies with regard to the given level of resistance of each FRC is lacking [36].

Recently, different studies have addressed this problem to close this gap in knowledge by conducting field experiments and focused on the evaluation of DM infections with regard to distinct amounts of PPTs applied [37,38]. As a result, it was found that PPTs can be reduced by up to 75% without affecting grape health [38]. Casanova-Gascón et al. [37] reported that a complete omission of PPTs was possible without negative effects on yield or quality in years with low DM infection pressure. However, omission of PPTs bears the risks of FRC infections under high pathogen pressure and the obviously devastating emergence of resistance-breaking isolates [38].

As further investigations were needed to develop specific PPT management guidelines for various FRCs, the present study included FRCs with different combinations of *R* loci against DM and PM to reveal their performance under field conditions. The impact of reduced PPT applications was investigated in consideration of both mildew pathogens, and results were compared to a range of susceptible cultivars that were co-located within the same vineyard as the FRCs, which allowed an evaluation under the same disease pressures. In addition, the economic benefit of the reduced number of applications at the farm level was specified, including four wineries.

The results of this study demonstrate the potential of FRCs to highly reduce fungicide applications and financial costs in viticulture. This knowledge and the developed specific PPT management strategies for FRCs could facilitate the increased cultivation of FRCs and therefore lead to a reduced use of fungicides in viticulture.

## 2. Results

### 2.1. Impact of Seasonal Climatic Conditions on Pathogen Infection Pressure and Resistance Level of FRCs

Annual climatic conditions determine the probability of infections caused by DM and PM and, thus, not only the resulting infection pressure but also the number of applications required.

**Figure 1:**
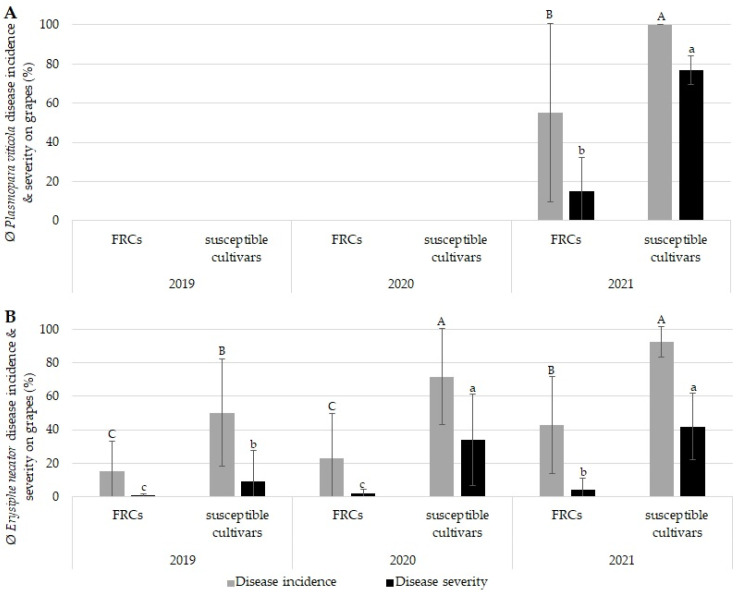
*Plasmopara viticola* and *Erysiphe necator* disease incidence and severity on grapes in different years (2019–2021). Shown are the average incidence (gray bar) and severity (black bar) of infection in fungus-resistant cultivars (FRCs) (‘Cabernet Blanc’, ‘Calardis Blanc’, ‘Muscaris’, ‘Satin Noir’, and ’Sauvignac’) and susceptible cultivars (‘Sauvignon Blanc’, ‘Cabernet Sauvignon’, ‘Riesling’, and ‘Muskateller’), in the untreated control. *P. viticola* disease incidence and severity (**A**) and *E. necator* disease incidence and severity (**B**) on grapes (BBCH 85) are shown. Bars show mean values of severity and incidence of infection on grapes (2019: n = 100; 2020: n = 75; 2021: n = 100). Error bars show standard deviation (stdev). Kruskal–Wallis and Conover–Iman tests were used for statistical data analysis to determine significance of disease incidence (A–C) or disease severity (a–c) between cultivars and different years; *p* < 0.05.

**Figure 2:**
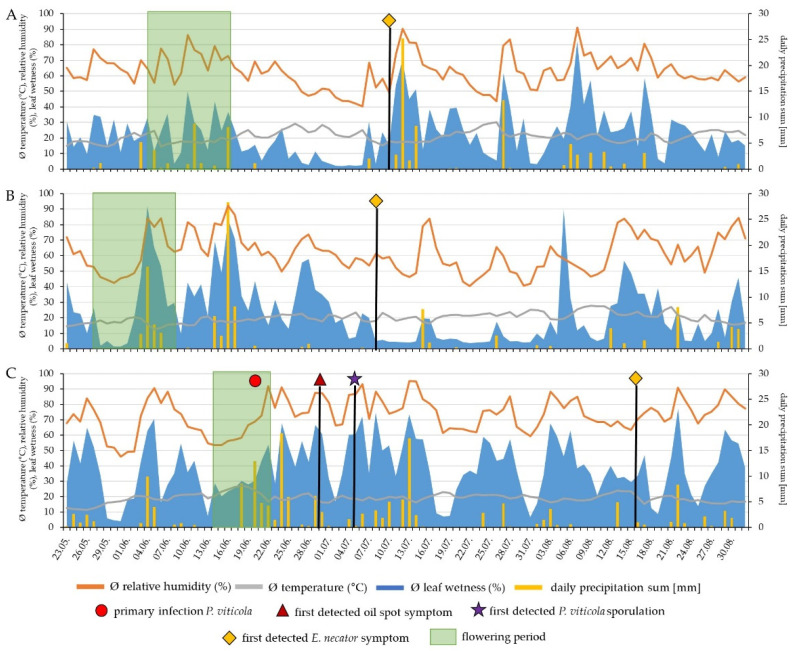
Weather conditions, *P. viticola* and *E. necator* infections in 2019–2021 (period from 23/05–31/08). Shown are daily mean temperature, relative humidity, leaf wetness, and total daily precipitation. (**A**) Data from 2019. (**B**) Data from 2020. (**C**) Data from 2021. Leaf wetness (%) is shown in light blue, precipitation total per day (mm) as yellow bars, relative humidity (%) in orange, daily average temperature (°C) in gray, first *E. necator* infections were marked with a yellow diamond, first oil spots are indicated with a dark red triangle, *P. viticola* primary infections by a light red circle, and first *P. viticola* sporulation on berries and leaves by a purple star. Bloom periods are highlighted in green, and show the flowering period for all cultivars and lasted (**A**) 5–7 days, (**B**) 10–12 days, and (**C**) 4–5 days. In 2019, flowering of the different cultivars started at different time points, while in 2020 and 2021, all cultivars were in bloom nearly at the same time.

To evaluate the impact of climatic conditions on PM and DM infection rates, the disease levels of different untreated FRCs and susceptible cultivars were assessed in a three-year field trial. Major annual differences in the disease levels of *P. viticola* and *E. necator* (Figure 1) strongly correlated with the distinct weather conditions observed in 2019, 2020, and 2021 (Figure 2).

In the years 2019 and 2020, no DM infection was detected on either the susceptible cultivars or the FRCs (Figure 1A). In contrast, 2021 was characterized by high DM disease pressure, with a primary *P. viticola* infection that was observed early during the season and resulted in significant DM infection of untreated FRCs and susceptible cultivars (Figure 1A, Figure 2C and Figure S1A). Significant differences in average DM disease incidence (DI) and severity (DS) were observed on grapes and leaves of untreated FRCs and susceptible cultivars (Figure 1A and Figure S1A). The DI on grapes of FRCs was about 55%, being significantly lower compared with that on susceptible cultivars, which showed a DI of 100% (Figure 1A). This difference was also reflected in a DS on grapes of approx. 15% in the case for FRCs compared with the 77% observed in susceptible cultivars (Figure 1A). The DS and DI on leaves were lower than on grapes with a DI of 40% (FRCs) and 98% (susceptible cultivars) and a DS of 4% (FRCs) and 18% (susceptible cultivars) (Figure 1A and Figure S1A).

In contrast with DM, PM infections were detected on all cultivars in all three years (Figure 1B and Figure S1B). In all years, the average PM infections (DI and DS) of untreated FRCs were significantly lower than those of untreated susceptible cultivars (Figure 1B and Figure S1B). Interestingly, PM infection in 2021 was observed much later but was significantly higher in FRCs than in 2019 and 2020 (Figure 1B and Figure 2). In 2021, PM infections mainly occurred on single berries, and only low DI and DS were observed on untreated leaves (Figure 1B and Figure S1B). In contrast to 2021, berries and leaves were comparably infected by *E. necator* in the years 2019 and 2020 (Figure 1B and Figure S1B). A detailed consideration of the prevalent weather conditions and plant and pathogen development is mandatory to understand the infection scenario in each year; therefore, the bioclimatic conditions of the three years are described in detail.

In 2019, the flowering period lasted 5–7 days in a cultivar-dependent manner over a period of 12 days (Figure 2A). During this period, rainfall occurred but not leading to infections with *P. viticola*. A hot and dry period followed, and *E. necator* started to spread thereafter, which resulted in colonization of leaves/berries first detected on 10/07/2019. The risk of infection decreased with berry development and temperature changes, resulting in limited disease severity (DS) for bunches. This resulted in 2019 in DIs of 14.9% (FRCs) and 50.1% (susceptible cultivars) and DSs of 0.51% (FRCs) and 9.3% (susceptible cultivars) (Figure 1B).

In 2020, the flowering period lasted twice as long as in 2019 and 2021 due to cooler temperatures (Figure 2B). During flowering, there was a risk of *P. viticola* infection due to the precipitation and air humidity but which did not manifest in any infection events (Figure 1A and Figure 2B). The first *E. necator* infection was observed in the beginning of July, and significant differences in the DS were observed between untreated FRCs and susceptible cultivars (Figure 2B and Figure 1B).

Due to warm temperatures, the flowering period in 2021 was rapid, lasting only 4–5 days, depending on the cultivar (Figure 2C). A continuous period of rainfall that began in mid-June resulted in a long period of leaf wetness and high humidity, which produced optimal conditions for primary *P. viticola* infections (Figure 2C). Five to eight days after primary infection, first oil spots were recognized; a few days later, the first sporulation was detected, which led to secondary infections (Figure 1A, Figure S1A and Figure 2C). As the berries continued to develop, the risk of infection steadily decreased, and the infected berries dried up. On the foliage, however, further secondary infections were produced due to morning dew and recurrent rainfall.

The first *E. necator* infection was observed at the end of July (Figure 1B and Figure 2C).

In general, the DI and DS of both pathogens on grapes were significantly higher on FRCs in 2021 than in 2019 and 2020, while FRCs had significantly lower DI and DS on grapes and leaves in all three years compared with susceptible cultivars (Figure 1).

### 2.2. Fungicide Saving Potential of FRCs against P. viticola in Field Trials

In 2021, optimal conditions for *P. viticola* infections were present. Field trials on the potential for fungicide savings were carried out in an experimental vineyard on the FRCs ‘Sauvignac’ (*Rpv12/3-1*), ‘Cabernet Blanc’ (*Rpv3-1*), ‘Calardis Blanc’ (*Rpv3-1/Rpv3-2*), ‘Satin Noir’ (*Rpv3-1*), and ‘Muscaris’ (*Rpv10*), as well as the susceptible cultivars ‘Sauvignon Blanc’, ‘Cabernet Sauvignon’, ‘Riesling’, and ‘Muskateller’.

To determine the potential fungicide savings, FRCs in the experimental vineyards were sprayed with a range of reduced crop-protection treatments (0, 2, and 4 treatments); and the *P. viticola* DI and DS were compared with those observed on susceptible cultivars within the same experimental vineyard that were treated using normal commercial PPT protocols (2021: 0 or 14 applications).

**Figure 3:**
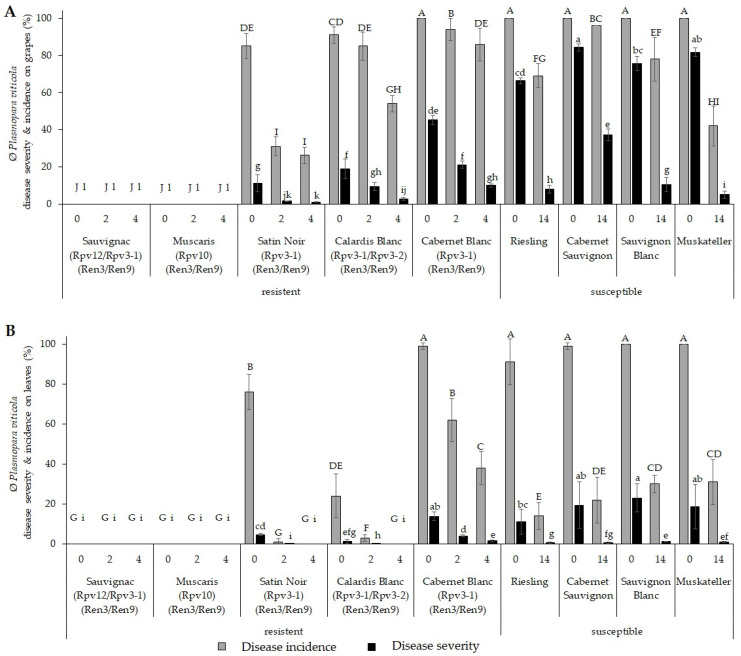
*Plasmopara viticola* disease incidence and severity on grapes and leaves of the experimental vineyard in 2021. Disease incidence (grey bars) and disease severity (black bars) in the fungus-resistant cultivars ’Sauvignac’ (*Rpv12*/*Rpv3-1*/*Ren3/Ren9*), ‘Muscaris’ (*Rpv10*/*Ren3/Ren9*), ‘Satin Noir’ (*Rpv3-1*/*Ren3/Ren9*), ‘Calardis Blanc’ (*Rpv3-1*/*Rpv3-2*/*Ren3/Ren9*), and ‘Cabernet Blanc’ (*Rpv3-1*/*Ren3/Ren9*) and the susceptible cultivars ‘Riesling’, ‘Cabernet Sauvignon’, ‘Sauvignon Blanc’, and ‘Muskateller’, each differing in the number of fungicide treatments during a season (0, 2, 4, or 14). (**A**) Shown are the mean values of disease severity and disease incidence at BBCH 83 on grapes (n (grapes) = 100 per variant). (**B**) Mean values of disease severity and incidence at BBCH 85 on leaves (n (leaves) = 100 per variant). Error bars show standard deviation (stdev). Kruskal–Wallis and Conover–Iman tests were used for statistical data analysis to determine significant differences in disease incidence (A–J) and disease severity (a–l) between cultivars and different fungicide treatments; *p* < 0.05.

The results showed that *P. viticola* infections were absent on the grapes and leaves of the FRCs ’Sauvignac’ (*Rpv12/Rpv3-1*) and ‘Muscaris’ (*Rpv10*) in any of the reduced crop protection treatments (Figure 3A). In contrast, *P. viticola* infections were detected on all susceptible cultivars, with a significantly lower extent on the *Rpv3-1* cultivars ‘Cabernet Blanc’ and ‘Satin Noir’ as well as on the *Rpv3-1/*Rpv3-2** cultivar ‘Calardis Blanc’ across all treatments (Figure 3). Interestingly, the grapes and leaves of the *Rpv3-1* cultivar ‘Satin Noir’ and the *Rpv3-1/*Rpv3-2** cultivar ‘Calardis Blanc’ showed significantly lower DI and DS compared with those of the *Rpv3-1* cultivar ‘Cabernet Blanc’ (Figure 3).

For susceptible cultivars, 14 treatments were necessary to accomplish a tolerable DS of ≤10% (except for ‘Cabernet Sauvignon’), whereas for ‘Calardis Blanc’ and ‘Satin Noir’, 2 treatments reduced the DS to ≤10% (Figure 3A). With 2 PPTs, the DI and DS on ‘Cabernet Blanc’ grapes and leaves was reduced significantly to 21.2% (DS, grapes), but four treatments were necessary to achieve comparable DS and DI to the susceptible cultivars of ≤10%, which were treated 14 times (Figure 3A). Compared with the 14 treatments in the susceptible cultivars, this corresponded to fungicide savings of ~71% for ‘Cabernet Blanc’ (4 PPTs) and ~86% for ‘Calardis Blanc’ and ‘Satin Noir’ (2 PPTs).

The 9.5% DS after 2 treatments in ‘Calardis Blanc’ and 1.5% DS in ‘Satin Noir’, was further reduced with 2 additional treatments in both cases. Thus, the DS for ‘Calardis Blanc’ after 4 treatments was significantly reduced to 2.6% and for ‘Satin Noir’ to 1.12% (Figure 3A). With the maximum level of 14 PPTs, it was not possible to obtain these low DS levels of <3% for any of the tested susceptible cultivars.

For grapevine leaves, a significantly lower DS was observed after 2 treatments for ‘Satin Noir’ (0.03%) and ‘Calardis Blanc’ (0.09%), compared with susceptible cultivars after 14 treatments (Figure 3B). For ‘Cabernet Blanc’, 4 treatments were necessary to accomplish a DS of 4.0% (on leaves), which was comparable to the DS of susceptible cultivars leaves treated 14 times.

### 2.3. Fungicide Saving Potential of FRCs against E. necator in Field Trials

**Figure 4:**
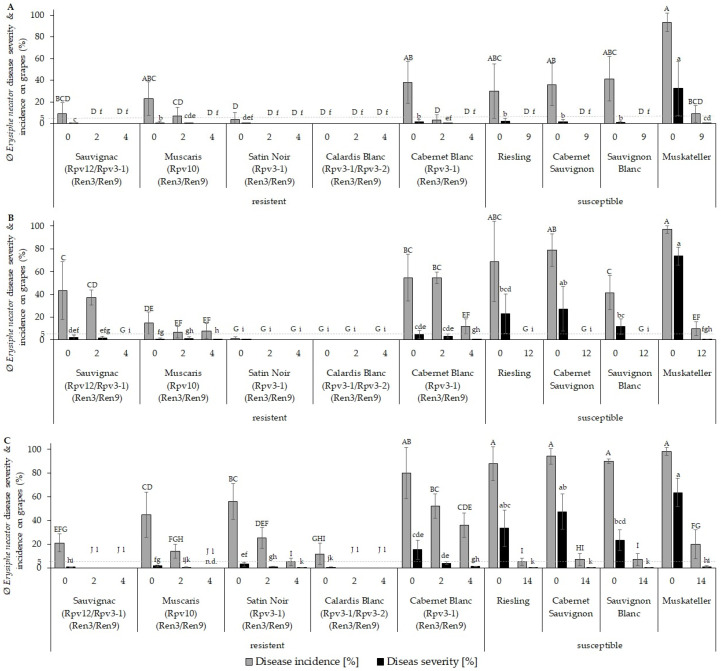
*Erysiphe necator* disease incidence and severity on grapes of the experimental vineyard in (**A**) 2019, (**B**) 2020, and (**C**) 2021. Disease incidence (grey bars) and disease severity (black bars) in the fungus-resistant cultivars ’Sauvignac’ (*Rpv12/Rpv3-1/Ren3/Ren9*), ‘Muscaris’ (*Rpv10/Ren3/Ren9*), ‘Satin Noir’ (*Rpv3-1/Ren3/Ren9*), ‘Calardis Blanc’ (*Rpv3-1/*Rpv3-2*/Ren3/Ren9*), and ‘Cabernet Blanc’ (*Rpv3-1/Ren3/Ren9*) and the susceptible cultivars ‘Riesling’, ‘Cabernet Sauvignon’, ‘Sauvignon Blanc’, and ‘Muskateller’, each differing in the number of fungicide treatments during a season (0, 2, 4; 9/12/14). Mean values of disease severity and incidence at BBCH 83 on grapes (n (grapes) = 100 per variant). Error bars show standard deviation (stdev). Kruskal–Wallis and Conover–Iman tests were used for statistical data analysis to determine significance of disease incidence (A–J) or disease severity (a–l) between cultivars and different fungicide treatments; *p* < 0.05.

During all years (2019–2021), *E. necator* infections were detected in all vineyards under investigation (Figure 1B, Figure 4, Figures S1 and S2). Experiments were performed as described above (Section 4.2) with a range of reduced plant-protection treatments for FRCs and susceptible cultivars. *E. necator* disease incidence and severity were evaluated and compared for FRCs (0, 2, and 4 applications) and susceptible cultivars (2019: 0 and 9 applications, 2020: 0 and 12 applications, and 2021: 0 and 14 applications) within the same vineyard.

PM infection pressure in 2020 and 2021 was higher than in 2019, as indicated by the high DS of the untreated susceptible cultivars of between 5 and 74% (Figure 4). In contrast to susceptible cultivars, the untreated FRCs showed a DS ≤ 5% in all experimental years, with the exception of ‘Cabernet Blanc’ in 2020 and 2021. In 2020 and 2021, 4 treatments were necessary for ‘Cabernet Blanc’ to obtain a DS comparable to that of susceptible cultivars treated according to the respective PPT protocol (12 or 14 treatments), allowing savings of ~67–71% of PPTs. Apart from the FRC ‘Cabernet Blanc’, 0 to 2 PPTs were sufficient for the other FRCs in all years to achieve high-quality grapes in terms of the grape health status, without significant DS differences compared with commercially treated susceptible cultivars (Figure 4). Compared with susceptible cultivars, the fungicide saving potential of these FRCs was therefore 78–100%.

For grapevine leaves, PM infections were, in all FRCs, lower than in grapes, apart from ‘Sauvignac’ (Figure S2).

### 2.4. Analysis of Cost Savings and Environmental Benefits Associated with the Use of FRCs

Since the number of PPTs could be reduced significantly without negative effects on plant or grape health when using FRCs (Figure 3 and Figure 4), costs for grapevine growers and environmental impacts can be reduced due to the significantly reduced amount of copper (Cu) and sulfur (S) applied. In all years, the amount of Cu used to protect FRCs was 0.42–0.50 kg/ha for 2 applications and 0.77–0.90 kg/ha for 4 PPTs (Table 1). In contrast, treatments of susceptible cultivars according to standard commercial practice (2019: 9 applications, 2020: 12 applications, and 2021: 14 applications) led to 1.73–1.81 kg/ha of Cu in the years 2019 and 2020, which had low DM disease pressure, and increased to 2.35 kg/ha Cu in the year in 2021 due to high disease pressure (Table 1). Thus, in 2019 and 2020, 2 PPTs were sufficient to protect FRCs from DM infections, which represents a copper reduction of 72% to 76% compared with that of susceptible cultivars. Even in 2021, 2–4 PPTs were adequate to protect FRCs from DM infections, which represents a Cu reduction of 62–79% compared with that of susceptible cultivars grown under the same conditions. The amount of pure S used in the treatments of FRCs was lower all years by approximately 8 kg/ha for 2 PPTs and approximately 12 kg/ha for 4 PPTs (Table 1) compared with approx. 23–31 kg/ha S for susceptible cultivars (Table 1). In most cases, only 2 PPTs for FRCs were necessary to maintain healthy plants and grapes, which represents an S reduction of 65–74% compared with susceptible cultivars grown under the same conditions. In 2020 and 2021, the FRC ‘Cabernet Blanc’ required 4 PPTs against PM, which still represents a ~60% reduction in S application.

Reducing the number of PPTs not only reduces the environmental impact but also the costs. To evaluate cost savings in more detail, the plant protection management of four wineries (two conventional working and two ecological working wineries) was evaluated over two years.

Each winery was able to reduce the number of PPTs required to control DM on FRCs compared with traditional cultivars. The wineries applied 10 to 15 PPTs for DM control on susceptible cultivars in 2021 and 8–11 PPTs in 2022 (Table 2 and Table 3). In contrast, FRCs only required 3–6 PPTs in 2021 and 2–5 PPTs in 2022 (Table 2 and Table 3). This represents a reduction in PPTs of 57–79% in 2021 and 50–75% in 2022 (Table 2 and Table 3). For the two ecologically working wineries, which exclusively used Cu-containing PPPs, the savings of Cu was calculated: 42% to 73% in 2021 and 52% to 59% in 2022 of Cu was saved when comparing FRCs to susceptible cultivars (Table 2).

A reduced number of PPTs required for DM control on FRCs also results in reduced costs linked with sprayer use, fuel, and working hours. The reduction in working hours was larger in the year with high DM infection pressure (2021), as the susceptible cultivars had to be treated to a greater extent. In this year, between 56% and 76% of the hours could be saved (Table 2). The savings in the year 2022, in which susceptible grapevine cultivars also required less plant protection due to weather conditions, were very different in the individual wineries. Thus, the savings in working hours were between 36% and 72% (Table 3). A reduction in working hours translates evidently into enormous savings in costs.

Taken together, the reduction in PPTs and working hours in FRCs resulted in strongly reduced costs compared with those of susceptible cultivars in both conventionally and ecologically working wineries. In 2021, wineries reduced their costs for plant protection management from about EUR 970–1400 for susceptible cultivars to EUR 310–670, thereby achieving a cost reduction with FRCs of 50% to 75% compared to their susceptible cultivars (Table 2). Comparable results were obtained in the following year, with a 46% to 72% cost reduction for the plant protection management of FRCs (Table 3).

## 3. Discussion

Sustainability evaluations of disease control strategies through experimental field studies are poorly documented. Therefore, different crop protection strategies based on reduced fungicide applications on fungus-resistant cultivars (FRCs) were evaluated in this study to determine the minimum spray regime required to ensure healthy grapes.

Weather conditions play an important role during the infection process. The infection of *P. viticola* requires wet and warm climatic conditions with a relative air humidity > 95%, temperatures between 13 and 19 °C, and leaf wetness levels above 90% [2,5,39]. In all three experimental years, these conditions for DM infection periods, with high precipitation, sufficient relative humidity, and leaf wetness levels, were present during flowering (Figure 2). Despite this, only in 2021 were high DM disease incidence and severity observed (Figure 1A). The discrepancy in DM infection pressure among the years could be attributed to the differences in the consecutive number of precipitation events during the infection period of the experimental years, as previous findings suggested that successive rainfalls events favor DM infections [40]. In 2019 and 2020, only a maximum of four consecutive days of rainfall were present during flowering; in 2021, six consecutive days of rain events occurred (Figure 2C). Furthermore, since only the daily mean values for temperature, precipitation, relative humidity, and leaf wetness levels were evaluated in this study, it is possible that, in 2021, diurnal variations in infection conditions had a pivotal impact on the high DM infections in 2021 alone. In contrast to *P. viticola*, *E. necator* requires much lower humidity (>40%) and a wide range of temperatures (10–32 °C) to allow germination of the conidia and infection [3,41,42]. As a result, PM infections were present in all trial years (Figure 1B).

Based on the absence of DM infections in untreated controls in 2019 and 2020 (Figure 1A), it appears that PPPs are not necessary for protection against DM infection in years with low infection pressure. However, the omission of PPT is not a recommendation for practice because of the possible unreliability of climatic prediction and the selection for resistance-breaking DM and PM isolates, which have emerged in FRC vineyards when PPTs are omitted and the levels of pathogen inoculum are allowed to increase [31,32,33,35,38,43]. The emergence of such resistance-breaking isolates and the erosion of plant resistance have also been described for apple and potato [44,45,46].

Wingerter et al. [38] examined the PPT-saving possibilities for the cultivars ‘Cabernet Blanc’ and ‘Sauvignac’, which were confirmed in the present study (Figure 3). In addition to this previous study, FRCs ‘Muscaris’ (*Rpv10*), ‘Satin Noir’ (*Rpv3-1*), and ‘Calardis Blanc’ (*Rpv3-1*/*Rpv3-2*) were examined, and the different impact of the various *R* loci of FRCs on the respective fungicide savings potential was documented. The results clearly showed, for *P. viticola* infections in 2021, the higher fungicide-saving potential of the cultivars ‘Sauvignac’ with the *Rpv12*/*Rpv3-1* loci and the *Rpv10* cultivar ‘Muscaris’, as infections were absent for untreated controls; with 10–50% disease severity (DS) for *Rpv3-1*, *Rpv3-1*/*Rpv3-2* cultivars, a lower fungicide-saving potential was observed (Figure 3). The differences in *Rpv12*-, *Rpv10*-, and *Rpv3*-mediated fungicide-saving potential observed in the field studies mostly correlated with the respective resistance level determined in laboratory experiments via leaf disc assays and field studies [32,38,47,48,49]. In contrast to laboratory experiments, stacking the two *Rpv3* loci did not appear to increase resistance in field, as the cultivar ‘Satin Noir’ (*Rpv3-1*) had significantly lower infection levels than ‘Calardis Blanc’ (*Rpv3-1*/*Rpv3-2*) (Figure 3) [34]. This issue was also highlighted by Zini et al. [49], whose results indicated that *R* loci pyramiding does not necessarily increase the disease resistance level against DM but may improve the durability of resistance. Surprisingly, ‘Satin Noir’ showed higher field resistance than ‘Cabernet Blanc’, even though both cultivars exclusively have the *Rpv3-1* locus. These resistance differences in the field could be provoked by physiological differences such as flowering duration or the density of vine canopies, which have been shown to influence DM infection [50].

Regarding plant protection recommendations, these differences imply that in years with high *P. viticola* infection pressure, cultivars with the *Rpv3* locus required between 2 and 4 PPTs, while cultivars with the *Rpv10* or *Rpv12* locus did not require any plant protection. However, the complete omission of PPTs is not a recommendation for practice due to the maintenance of resistance, as explained previously.

Our results also demonstrate that the complete omission of PPTs can lead to significant DM infection in FRCs that rely exclusively on *Rpv3*-mediated resistance. This is in contrast to the findings of Casanova-Gascón et al. [37], who observed no negative effects caused by the omission of plant protection for FRCs, possibly because of the lower infection pressure in their field trails. The presented study highlights that the resistance loci present in a FRC have to be considered for optimal plant protection management to prevent risking negative effects on plant and grape health and to achieve the maximal saving of fungicides. However, the results also show that with the used FRC cultivars, a minimum 71% reduction in PPTs against DM was possible in all experimental years. The achieved results show the high fungicide-saving potential of FRCs and demonstrate the advantage and need of the pyramidization of different *R* loci in new FRCs for a higher degree and sustainability of resistance against DM [16,51].

While grape infections can have severe effects on yield and grape quality, studies indicated that canopy infections, to a certain extent, have little impact on yield and quality and can be compensated for by the plant [52,53]. In the present study, infections of leaves were low even in the untreated susceptible cultivars (Figure 3B). Except for ‘Cabernet Blanc’, 2 treatments in the FRCs and 14 treatments in the susceptible cultivars were sufficient to maintain an infection severity below 2%, which probably had no effect on plant growth, yield, or quality (Figure 3B).

Assessment of the performance of FRCs in terms of susceptibility to PM over three years of field experiments demonstrated that, under most climatic conditions, 2 PPTs were sufficient to protect grapes of FRCs from PM infections and produce grapes of similar or better health than traditional susceptible cultivars that had been sprayed between 9 and 14 times (Figure 4). To the best of our knowledge, this is the first study evaluating the effects of fungicide savings concerning PM infections in FRCs. In addition to the possibility of reducing the number of PPTs, it is evident that the amount of S applied in the vineyard to control PM can be significantly reduced with the use of FRCs.

The level of PM DS on grapes of all FRCs apart from ‘Cabernet Blanc’ was below 3% in untreated and ≤1.5% after 2 PPTs (Figure 4). For the widely used grapevine cultivars like ‘Riesling’, ‘Chardonnay’, or ‘Cabernet Sauvignon’ a threshold level that causes off-flavors in wine was shown to be 1–5% [54,55]; even though experimental data for FRCs are missing, similar thresholds levels for off-flavors can be expected for FRCs. This indicates that negative effects on grape or wine quality by a reduction in PPTs with FRCs are very unlikely.

However, it should be noted that while the FRCs examined in these field trials contained different combinations of *R* loci against DM, they all contained the same combination of *R* loci against PM, designated the *Ren3* and *Ren9* loci [26]. The observed differences in resistance against PM of FRCs, with the *Ren3/Ren9* locus, could be due to additional unknown *R* loci or due to physiological differences [20,49]. The general physiological differences in FRCs were not evaluated in this study, so there could have been differences in the densities of the vine canopies and compactness of the grape clusters, which favor PM development by decreasing air circulation, light penetration, and prohibit effective application of fungicides [50]. Furthermore, the thickness and composition of epicuticular waxes, the cuticle, the epidermis, and the hypodermis could also have an influence on susceptibility, since they act as protective barriers against pathogens [56,57]. As was the case for DM, leaf infections were negligible for PM, as neither wood maturation nor photosynthetic performance were likely to be compromised by the infections (Figure S2).

Compared with the susceptible cultivars, the number of PPTs could be reduced between 67 and 100% considering DM and PM treatments, resulting in the usage of ≤0.9 kg Cu and 12 kg S per ha without affecting plant or grape health. However, the fungicide-reduction potential depended on the cultivar and on climatic conditions (Figure 3 and Figure 4). The results demonstrated that the use of FRCs allows safe production with markedly reduced copper and sulfur amounts, below the limit of 4 kg of Cu per hectare set by the European Union and below the limit of 3 kg/ha set by the German organic growers’ associations under field conditions [15,58,59,60,61]. The use of FRCs combined with an adopted plant protection strategy allows reductions of Cu and S by ≥50%, which meet the criteria of the European Green Deal to reduce the number of PPPs by 50% by 2030 (Table 1) [11]. In addition, the use of FRCs reduces costs related to plant management, such as machine use and staff resources, by 46 to 75% compared with those of susceptible cultivars (Table 2 and Table 3). In addition to the financial savings, FCRs can save up to 76% of the time spent on plant protection management. Prices for pesticides are skyrocketing, their availability is becoming more restricted, and additional labor and machine costs for spraying are putting further strain on wineries’ costs when the profitability of wine estates is already jeopardizing the long-term existence of wine estates [62]. The reduction of PPTs via the use of FRCs can reduce soil compression, as a result of repeated treatments with heavy devices, which lead to restricted root development and may have negative effects on grape quality and yield [63,64,65].

Taken together, this study highlights that in addition to the genetically determined resistance levels, other factors such as climatic conditions and the prevailing pathogen and viticultural factors play a role in the absolute resistance of a cultivar and, thus, define the number of fungicide treatments required. Compared with susceptible cultivars, PPTs against both mildew pathogens were reduced ≥67% without negative effects on plant or grape health, depending on the cultivar, pathogen, and climatic conditions. This shows that FRCs can be an important tool to reduce PPTs in viticulture, which will lead to significant reductions in the cost of grape production and dramatically reduce the environmental impact of viticulture.

## 4. Materials and Methods

### 4.1. Grapevine Cultivars Evaluated in Field Experiments and Infections with Downy and Powdery Mildew

Field experiments were conducted with the FRCs ‘Satin Noir’ (*Rpv3-1*/*Ren3*/*Ren9*) [38] (rootstock: Paulsen 1103), ‘Cabernet Blanc’ (*Rpv3-1*/*Ren3*/*Ren9*) (rootstock: SO4), ‘Calardis Blanc’ (*Rpv3-1*/*Rpv3-2*/*Ren3*/*Ren9*) (rootstock: SO4), ‘Muscaris’ (*Rpv10*/*Ren3*/*Ren9*) (rootstock: SO4), and ‘Sauvignac’ (*Rpv12*/*Rpv3-1*/*Ren3/Ren9*) (rootstock: 5BB) [26,27]. Susceptible cultivars evaluated in the experiments were ‘Cabernet Sauvignon’ (rootstock: SO4), ‘Muskateller’ (rootstock: SO4), ‘Riesling’ (rootstock: SO4), and ‘Sauvignon Blanc’ (rootstock: Couderc 3309). The combinations of graft cuttings to rootstocks were adjusted to the soil- and the cultivar-specific physiological characteristics (vegetative and generative growth) to guarantee comparable growth of all vines. Infections with *Plasmopara viticola* Berl. & de Toni and *Erysiphe necator* Schweinitz Braun & Takamatsu occurred naturally without any external intervention.

### 4.2. Experimental Sites

Experimental locations were in Rhineland-Palatinate, Germany. Experiments were conducted on an experimental vineyard planted in 2016 of the DLR Rheinpfalz (coordinates: 49.37193793096786, 8.182542958307094) on the FRCs ‘Sauvignac’, ‘Cabernet Blanc’, ‘Calardis Blanc’, ‘Satin Noir’, and ‘Muscaris’, as well as the susceptible cultivars ‘Sauvignon Blanc’, ‘Cabernet Sauvignon’, ‘Riesling’, and ‘Muskateller’. All cultivars evaluated were located in the same vineyard. A total of 135 vines are planted in rows (width: 2.05 m) with a planting distance of 1.1 m. All cultivars were cultivated with the vertical shoot positioned at the espalier. Vineyard management was performed identically for all cultivars and plots according to German ecological standards. Rows of FRCs and susceptible cultivars were randomly planted in the experimental field. The experimental vineyard was cultivated according to German ecological standards. Thus, the use of synthetic fungicides was prohibited, and only plant protection products containing copper and sulfur were utilized.

### 4.3. Reduced Fungicide Treatment

To evaluate the fungicide-saving potential of FRCs, reduced fungicide treatments were applied. Fungicide treatments were implemented according to a treatment plan [38]. The time point of treatment was predefined for grapevine development (BBCH-scale) according to Lorenz et al. [66]. Identical treatment variants were chosen for all FRCs. In treatment variant 4, the first treatment took place during early leaf development (BBCH 15), followed by 2 treatments during flowering, (BBCH 57–60 and BBCH 69), and a final treatment when berries reached pea-size (BBCH 75–77). The second variant was treated during flowering (BBCH 57–59 and BBCH 69), and an untreated variant was monitored as a control to classify and compare annual natural infection pressure. The susceptible cultivars were treated standardly, independent of the treatment plan.

The vines were distributed over four blots with 25 vines each. Per rating blot, 25 leaves and 25 grape clusters were evaluated randomly from both sides of the row. The location of variants differed between all experimental years and was repositioned annually.

### 4.4. Assessment of Disease Severity and Incidence

Downy and powdery mildew disease severity (DS) and incidence (DI) were concurrently evaluated weekly, starting with BBCH 14 (4th leaf unfolded). The diseases were identified according to the European Plant Protection Organization (EPPO) standards, and the disease severity scheme of EPPO was modified and used to assess disease severity [66,67]. To simplify the evaluation, only eight instead of twelve symptom grades were used: 0%, (no symptoms); ≤1%, <5%, <10%, <25%, <50%, <75%, and ≤100% infection.

Average DI and DS are shown with standard deviation. Averages were used for Kruskal–Wallis with Conover–Iman test to determine the effects of the different treatments on the DS and DI. The software used for statistical analysis was XLSTAT 2019.3.2, Add-On for Microsoft Excel^®^.

### 4.5. Weather Data

Weather data were collected using the weather station located within a 1 km radius of the experimental vineyard at the DLR Rheinpfalz in Neustadt an der Weinstraße. This station was provided and maintained by the Agrarmeteorologie Rhineland-Palatinate. The data were accessible online and were downloaded from the service of the providers (Agrarmeteorologie Rhineland-Palatinate). The sum of precipitation and averages of daily air temperature (2 m above ground), leaf wetness, and relative air humidity were used.

### 4.6. Economic Evaluations

Four wineries located in the two different German wine growing regions Rhinehessen (state Rhineland Palatinate) and Rheingau (state Hesse) participated in the study. All of the wineries harvested grapes from susceptible cultivars and FRCs. In sum, the four wineries possessed 389 hectares of vineyard, and 43 hectares were planted with FRCs, accounting for about 11% of their planting. Two winegrowers worked conventionally and two accordingly to organic standards. The plots were identified upfront in order to allow later analyses of FRCs versus susceptible plant treatments but also to avoid flexibility in later selection to eventually influence the analyses. Observed susceptible cultivars were ‘Riesling’, ‘White Burgundy’, ‘Pinot Noir’, ‘Scheurebe’, ‘Dornfelder’, ‘Müller-Thurgau’, ‘Gewürtztraminer’, ‘Chardonnay’, and ‘Acolon’. The FRCs observed consisted of ‘Calardis Blanc’, ‘Souvignier Gris’, ‘Sauvignac’, ‘Cabertin’, ‘Accent’, ‘Regent’, ‘Muscaris’, ‘Johanniter’, ‘Solaris’, ‘Pinotin‘, and ‘Prior’.

The study opted to apply the GPS-based tracking of vineyard activities, called Vinumcloud (Agrinovate Research & Development GmbH; Burrweiler, Germany). All wineries´ processes in the vineyard were tracked using a digital field index and process tracking. The partners signed agreements with the service provider, and software and service costs were born by the research grant. Then, all geodata for the identified plots of the wineries´ were fed into the system. Participants were trained in accurate data gathering. Digital tracking allowed the avoidance of eventual bias that could be expected when examining pesticide treatments. Full tracking was realized in 2021 and 2022. The software provided processes performed, time consumption, and all materials used. In step two, material costs were allocated based on real prices. For the performed tasks, costs were calculated based on the available data in the literature as benchmarks and industry averages [68] and from institutions advising German vintners and wineries. The third step consisted of a validation of all cost-allocation schemes by wine industry experts and by representatives of all observed wineries. Using these data, the total costs for PPTs were calculated. Total costs included PPP costs, employee costs (35 EUR/h) and machine costs (42 EUR/h). The bases for employee and machine costs were, in each case, the working hours, which were collected with Vinumcloud. The cost rates were set rather conservatively, meaning they were set at a low level.

## Figures and Tables

**Table 1:** Amounts of pure copper (Cu) and sulfur (S) applied to susceptible cultivars with standard plant protection treatment (PPT) strategies (2019: 9 applications, 2020: 12 applications, and 2021: 14 applications) and fungus-resistant cultivars (FRCs) with two or four PPTs in 2019, 2020, and 2021. Data obtained in the experimental field of the DLR Rheinpfalz, Germany.

	2019 PPTs			2020 PPTs			2021 PPTs		
	9	2	4	12	2	4	14	2	4
Amount of Cu (kg) per ha	1.81	0.50	0.81	1.73	0.42	0.77	2.35	0.50	0.90
Relative amount of Cu compared to standard PPTs per ha (%)	100	28	45	100	24	44	100	21	38
Amount of S (kg) per ha	22.85	8.00	12.00	30.83	8.00	11.98	29.61	7.96	11.94
Relative amount of S compared to standard PPTs per ha (%)	100	35	52	100	26	39	100	73	40

**Table 2:** Number of plant protection treatments (PPTs), amount of pure copper (Cu) applied, working hours, and costs of PPTs of susceptible cultivars (SCs) vs. fungus-resistant cultivars (FRCs) in 2021 in two conventional (conv.) and two ecological (eco.) working wineries. Vineyard activities were tracked using a GPS-based system. Using these data, total costs for PPTs were calculated. Total costs included PPP costs, employee costs, and machine costs. Basis for employee and machine costs are the working hours, which were collected with a GPS-based tracking system. FRCs, compared with susceptible cultivars, resulted in reductions in PPTs, Cu, working hours, and total costs.

	Winery 1 (conv.)		Winery 2 (conv.)		Winery 3 (eco.)		Winery 4 (eco.)	
	SCs	FRCs	SCs	FRCs	SCs	FRCs	SCs	FRCs
Number of PPTs	11	4	10	4	14	3–6	15	5
Amount of Cu (kg/ha)	-	-	-	-	3	0.8–1.75	3.7	1.3
Working hours (h/ha)	7.1	2.4	3.9	1.6	10.7	2.6	5.4	2.4
Total costs for PPTs (EUR/ha)	1438	472	1494	672	1232	311	964	478
Reduction of number of PPTs in FRCs (%)	64		60		57–79		67	
Reduction of Cu in FRCs (%)	-		-		42–73		65	
Reduction of working hours in FRCs (%)	66		59		76		65	
Reduction of total costs in FRCs (%)	67		55		75		50	

**Table 3:** Number of plant protection treatments (PPTs), amount of pure copper (Cu) applied, working hours, and costs of PPTs of susceptible cultivars (SCs) vs. fungus-resistant cultivars (FRCs) in 2022 in two conventional (conv.) and two ecological (eco.) working wineries. Vineyard activities were tracked using a GPS-based system. Using these data, total costs for PPTs were calculated. Total costs included PPP costs, employee costs, and machine costs. Basis for employee and machine costs were the working hours, which were collected with the GPS-based tracking system. FRCs, compared with susceptible cultivars, resulted in reductions in PPTs, Cu, working hours, and total costs.

	Winery 1 (conv.)		Winery 2 (conv.)		Winery 3 (eco.)		Winery 4 (eco.)	
	SCs	FRCs	SCs	FRCs	SCs	FRCs	SCs	FRCs
Number of PPTs	9	3–4	8	2–3	11	4	10	5
Amount of Cu (kg/ha)	-	-	-	-	1.6	0.65	1.24	0.6
Working hours (h/ha)	5.6	2.6	3.44	1.1	6.9	1.9	2.5	1.6
Total costs for PPTs (EUR/ha)	1002	541	692	233	827	231	477	233
Reduction of number of PPTs in FRCs (%)	56–67		63–75		64		50	
Reduction of Cu in FRCs (%)	-		-		59		52	
Reduction of working hours in FRCs (%)	54		68		73		36	
Reduction of total costs in FRCs (%)	46		66		72		51	

## Data Availability

The datasets supporting the conclusions of this article are included within the article and Supplementary Materials.

## Supplementary Materials

The following supporting information can be downloaded at: https://www.mdpi.com/article/10.3390/plants12173120/s1. Figure S1. *Plasmopara viticola* and *Erysiphe necator* disease incidence and severity on leaves in different years (2019–2021). Average incidence (gray bar) and severity (black bar) of infection in fungus resistant cultivars (FRCs) (‘Cabernet Blanc’, ‘Calardis Blanc’, ‘Muscaris’, ‘Satin Noir,’ and ’Sauvignac’) and susceptible cultivars (‘Sauvignon Blanc’, ‘Cabernet Sauvignon’, ‘Riesling’, and ‘Muskateller’), in the untreated control. *P. viticola* disease incidence and severity (A) and *E. necator* disease incidence and severity (B) on leaves (2019 BBCH 89; 2020–2021 BBCH 85) are shown. Bars show mean values of severity and incidence of infection on leaves (2019: n = 100; 2020: n = 75; 2021: n = 100). Error bars show standard deviation (stdev). Kruskal–Wallis and Conover–Iman tests were used for statistical data analysis to determine significance of disease incidence (A–C) or disease severity (a–c) among cultivars and different years; *p* < 0.05. Figure S2. *Erysiphe necator* disease incidence and severity on leaves in different years (A) 2019, (B) 2020, and (C) 2021. Disease incidence (grey bars) and disease severity (black bars) in the fungus-resistant cultivars ’Sauvignac’ (*Rpv12/Rpv3-1/Ren3/Ren9*), ‘Muscaris’ (*Rpv10/Ren3/Ren9*), ‘Satin Noir’ (*Rpv3-1/Ren3/Ren9*), ‘Calardis Blanc’ (*Rpv3-1/Rpv3-2/Ren3/Ren9*), and ‘Cabernet Blanc’ (*Rpv3-1/Ren3/Ren9*) and the susceptible cultivars ‘Riesling’, ‘Cabernet Sauvignon’, ‘Sauvignon Blanc’, and ‘Muskateller’, each differing in the number of fungicide treatments during a season (0, 2, 4, or 14). Mean values of disease severity and disease incidence on leaves (2019 BBCH 89; 2020-2021 BBCH 85). Bars show mean values of severity and incidence of infection on leaves (2019: n = 100; 2020: n = 75; 2021: n = 100). Error bars show standard deviation (stdev). Kruskal–Wallis and Conover–Iman tests were used for statistical data analysis to determine significant differences of disease incidence (A–J) or disease severity (a–l) among cultivars and different fungicide treatments; *p* < 0.05.
