# Supplementary figures and images for: Fungicide-Saving Potential and Economic Advantages of Fungus-Resistant Grapevine Cultivars
Source: Plants (Basel). 2023 Aug 30;12(17):3120. doi: 10.3390/plants12173120 (PMC10489737; doi:10.3390/plants12173120)

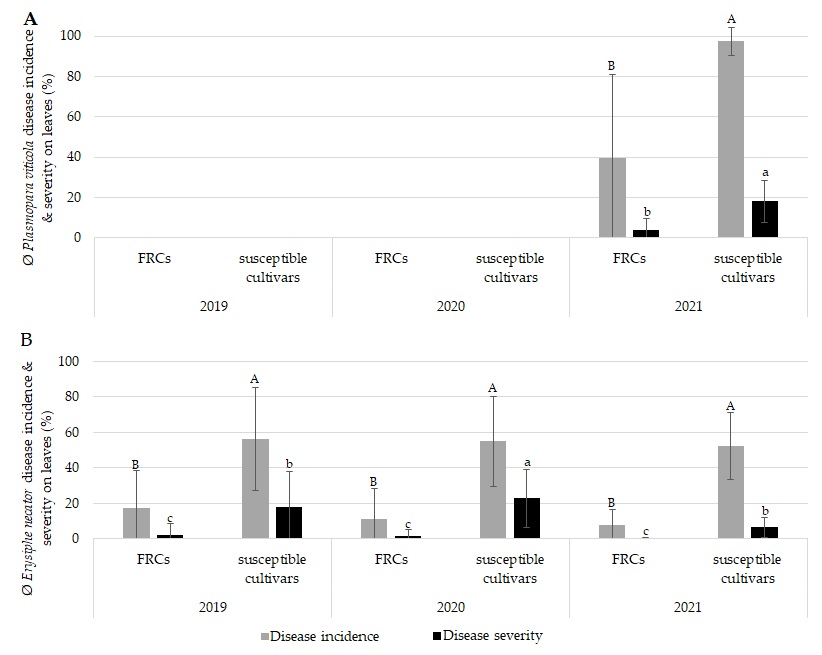

Supplement: Supplementary file 1 [file plants-12-03120-s001.zip › FigureS1.jpg]

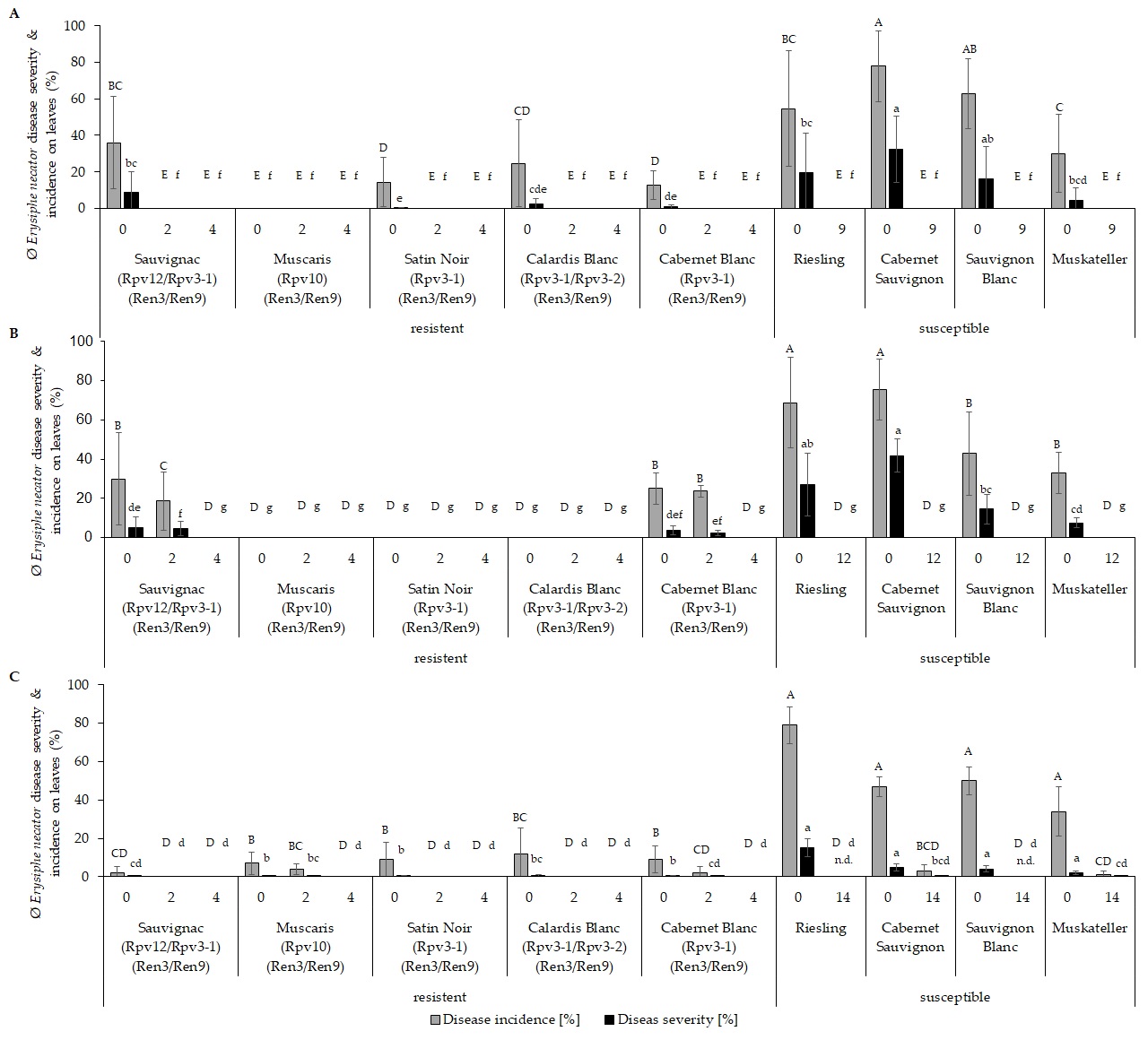

Supplement: Supplementary file 1 [file plants-12-03120-s001.zip › FigureS2.jpg]
